# Adherence to Perinatal Asphyxia or Sepsis Management Guidelines in Low- and Middle-Income Countries

**DOI:** 10.1001/jamanetworkopen.2025.10790

**Published:** 2025-05-16

**Authors:** Afruna Rahman, Meghna Ray, Zachary J. Madewell, Kitiezo Aggrey Igunza, Victor Akelo, Dickens Onyango, Florence Murila, Winnie Mwebia, Ikechukwu Udo Ogbuanu, Julius Ojulong, Dickens Kowuor, Erick Kaluma, Solomon Samura, Shams El Arifeen, Emily S. Gurley, Mohammad Zahid Hossain, Kazi Munisul Islam, Rajib Biswas, Nega Assefa, Temesgen Teferi, Konjit Eshetu, Lola Madrid, Karen L. Kotloff, Milagritos D. Tapia, Adama Mamby Keita, Elisio Xerinda, Celisa Mendonça de Assis, Milton Kincardett, Inacio Mandomado, Rosauro Varo, Shabir A. Madhi, Ziyaad Dangor, Vuyelwa Baba, Sithembiso Velaphi, Yasmin Adam, Dianna M. Blau, Portia C. Mutevedzi, Quique Bassat, Cynthia G. Whitney, Chris A. Rees

**Affiliations:** 1 International Centre for Diarrhoeal Disease Research Bangladesh (ICDDRB), Dhaka, Bangladesh; 2 Hubert Department of Global Health, Rollins School of Public Health, Emory University, Atlanta, Georgia; 3 Global Health Center, US Centers for Disease Control and Prevention, Atlanta, Georgia; 4 Kenya Medical Research Institute, Center for Global Health Research, Kisumu, Kenya; 5 Liverpool School of Tropical Medicine, Liverpool, United Kingdom; 6 Global Health Institute, Emory University, Atlanta, Georgia; 7 Kisumu County Department of Health, Kisumu, Kenya; 8 University of Nairobi, Nairobi, Kenya; 9 CHAMPS Project, Freetown, Sierra Leone; 10 World Hope International, Makeni, Sierra Leone; 11 Department of Epidemiology, Johns Hopkins Bloomberg School of Public Health, Baltimore, Maryland; 12 London School of Hygiene and Tropical Medicine, London, United Kingdom; 13 College of Health and Medical Sciences, Haramaya University, Harar, Ethiopia; 14 Department of Pediatrics, Center for Vaccine Development and Global Health, University of Maryland School of Medicine, Baltimore; 15 Centre pour le Développement des Vaccins-Mali, Bamako, Mali; 16 Centro de Investigação em Saúde de Manhiça (CISM), Maputo, Mozambique; 17 Hospital Central de Quelimane, Quelimane, Mozambique; 18 ISGlobal - Hospital Clínic, Universitat de Barcelona, Barcelona, Spain; 19 South African Medical Research Council Vaccines and Infectious Diseases Analytics Research Unit, Faculty of Health Sciences, University of the Witwatersrand, Johannesburg, South Africa; 20 Wits Infectious Diseases and Oncology Research Institute, Faculty of Health Sciences, University of the Witwatersrand, Johannesburg, South Africa; 21 Department of Obstetrics and Gynaecology, University of the Witwatersrand, Johannesburg, South Africa; 22 Department of Pediatrics, Chris Hani Baragwanath Academic Hospital, School of Clinical Medicine, Faculty of Health Sciences, University of the Witwatersrand, Johannesburg, South Africa; 23 Institución Catalana de Investigación y Estudios Avanzados (ICREA), Pg. Lluís Companys 23, Barcelona, Spain; 24 Department of Pediatrics, Hospital Sant Joan de Déu, Universitat de Barcelona, Esplugues, Barcelona, Spain; 25 Division of Pediatric Emergency Medicine, Emory University School of Medicine, Atlanta, Georgia

## Abstract

**Importance:**

Most of the 2.3 million annual neonatal deaths occur in sub-Saharan Africa and South Asia, with perinatal asphyxia and neonatal sepsis being the leading causes of neonatal mortality. Most neonatal deaths are considered preventable through high-quality clinical care, which includes adherence to clinical care guidelines.

**Objective:**

To assess adherence to World Health Organization clinical care guidelines for management of perinatal asphyxia and neonatal sepsis and to identify patient-level factors in adherence among neonates who died from these conditions.

**Design, Setting, and Participants:**

This cross-sectional study obtained data from December 2015 through October 2023 from the Child Health and Mortality Prevention Surveillance (CHAMPS) catchment areas in 7 low- and middle-income countries in sub-Saharan Africa (Ethiopia, Kenya, Mali, Mozambique, Sierra Leone, and South Africa) and South Asia (Bangladesh). Participants were neonates who were born alive and were aged 0 to 28 days at the time of death and had either perinatal asphyxia or neonatal sepsis.

**Exposure:**

Medical records of neonates who died from perinatal asphyxia or neonatal sepsis determined by postmortem diagnostics.

**Main Outcomes and Measures:**

The main outcome was the proportion of deceased neonates who received guideline-adherent treatments before they died. Mixed-effect multivariable logistic regression analyses were performed to identify factors associated with administration of at least bag-valve-mask (BVM) ventilation for perinatal asphyxia.

**Results:**

Of the 1194 neonates (median [IQR] age at the time of death, 2 [1-6] days; 692 males [58.0%]) who died and were enrolled in CHAMPS with available clinical data, 476 (39.9%) died from perinatal asphyxia, 562 (47.0%) died from neonatal sepsis, and 156 (13.1%) from both conditions. These neonates had a median (IQR) birth weight of 2130 (1266-2988) g. For cases with perinatal asphyxia, guideline adherence ranged from 12.2% (n = 77) for adrenaline administration to 85.4% (540) for supplemental oxygen administration. Only 4.4% of neonates (28) with perinatal asphyxia received all recommended treatments. Among cases with neonatal sepsis, antibiotics were administered to 86.8% (623), although the recommended treatment was administered to only 61.0% (438). In multivariable analyses, neonates in whom clinicians accurately identified perinatal asphyxia were more likely to receive BVM ventilation than those who had received discordant antemortem and postmortem diagnoses (adjusted odds ratio, 2.00; 95% CI, 1.29-3.12).

**Conclusions and Relevance:**

In this cross-sectional study, clinical care guideline adherence was suboptimal among neonates who died from perinatal asphyxia or neonatal sepsis. This finding underscores the critical need to increase adherence in regions with high rates of neonatal mortality and may inform strategies for strengthening health systems to support compliance with clinical care guidelines.

## Key Points

**Question:**

Do neonates who die of perinatal asphyxia or sepsis in low- and middle-income countries receive guideline-adherent clinical care?

**Findings:**

In this cross-sectional study of 1194 neonates who died, less than 5% of those who died with perinatal asphyxia received all recommended clinical care, and 61% of those who died from sepsis were given recommended antibiotics.

**Meaning:**

The suboptimal adherence to World Health Organization clinical guidelines underscores the need to increase adherence in regions with high rates of neonatal mortality.

## Teaser

This cross-sectional study examines clinicians’ adherence to the World Health Organization Pocket Book of Hospital Care for Children recommendations for hospitalized newborns who died with perinatal asphyxia, sepsis, or both conditions in sub-Saharan Africa and South Asia.

## Introduction

In 2021, there were 2.3 million deaths globally among neonates.^1^ Sub-Saharan Africa accounted for 45% (1.1 million) of neonatal deaths despite representing only 29% of live births.^1^ South Asia also bears a disproportionate burden of neonatal mortality, accounting for 35% (0.8 million) of neonatal deaths, despite representing only 27% of live births in 2021.^1^ Perinatal asphyxia and neonatal sepsis are among the leading direct causes of death among neonates, accounting for more than half of all neonatal deaths.^2,3^

Most neonatal deaths are considered preventable through timely and high-quality clinical care.^4,5,6,7^ Adherence to clinical care guidelines is a crucial component of high-quality care. Previous studies have shown varied adherence to clinical care recommendations, with few studies focusing specifically on neonates who died.^8,9,10,11,12,13^ However, as neonatal deaths account for nearly half of all deaths among children younger than 5 years, understanding clinical care guideline adherence in this population is essential in identifying gaps that could be targeted for increased survival.^1^

In this study, our objective was to assess adherence to World Health Organization (WHO) guidelines for the management of perinatal asphyxia and neonatal sepsis and to identify patient-level factors in adherence among neonates who died from these conditions. The study focused on neonate deaths in 7 countries in sub-Saharan Africa and South Asia (Bangladesh).^14^

## Methods

### Study Design and Setting

We conducted a cross-sectional study using clinical data of neonates who died. These deaths were identified through prospective childhood mortality surveillance at 7 sites within the Child Health and Mortality Prevention Surveillance (CHAMPS) network. Written informed consent to participate in CHAMPS was provided by caregivers. The Emory University Institutional Review Board and the ethics review boards at each of the study sites approved the use of CHAMPS data for this study. We followed the Strengthening the Reporting of Observational Studies in Epidemiology (STROBE) reporting guideline.

Established in 2015, CHAMPS systematically gathers standardized, population-based surveillance data from regions with high mortality rates in children younger than 5 years. The primary objective of CHAMPS is to determine causes of death to inform evidence-based interventions for improving child health outcomes.^15^ CHAMPS operates in catchment areas in 7 countries: Bangladesh (Baliakandi and Faridpur), Ethiopia (Kersa and Harar), Kenya (Kisumu and Siaya), Mali (Bamako), Mozambique (Manhiça and Quelimane), Sierra Leone (Makeni and Bo), and South Africa (Soweto and Thembelihle). Surveillance activities are conducted in national referral hospitals, district hospitals, and community settings to provide a representation of childhood deaths (eTable 1 in Supplement 1).

### Data Sources

After identification of a death, CHAMPS staff approach caregivers of the stillbirth or deceased neonate, infant, or child to solicit written informed consent to participate in CHAMPS. Deaths identified within 24 hours (or ≤72 hours if the body has been refrigerated) are eligible for minimally invasive tissue sampling (MITS), clinical record review, and verbal autopsy. Trained CHAMPS clinical staff systematically review available medical records, including admission records, daily progress notes, laboratory and radiologic results, and hospital registries, to extract detailed clinical data using standardized abstraction forms.^16^ CHAMPS staff also conduct verbal autopsies; gather demographic data; and conduct MITS, which provides microbiological and histopathologic information for each deceased child.^16^ At each CHAMPS site, causes of death are ascertained by a Determination of Cause of Death (DeCoDe) panel, which includes local clinicians, pathologists, and public health professionals, following the *International Statistical Classification of Diseases and Related Health Problems, Tenth Revision*.

### Inclusion and Exclusion Criteria

This study was restricted to neonates (born alive and aged 0-28 days at the time of death) who were hospitalized prior to and at the time of death, were enrolled in CHAMPS, underwent MITS, and died from perinatal asphyxia or neonatal sepsis as determined by a DeCoDe panel. Along with an evaluation of clinical, verbal autopsy, microbiological, and histopathologic data,^16^ perinatal asphyxia cases were defined as those with evidence of hypoxia as a cause of death and neonatal sepsis cases were defined as those who died from a systemic infection.

Because the precise time of asphyxia (ie, intrapartum or postnatal) could not be distinguished, all cases of neonates born alive and found to have evidence of perinatal asphyxia were included. All neonates who died with either condition (perinatal asphyxia or neonatal sepsis) in any position in the causal chain of death (ie, immediate, underlying, or comorbid) were included from December 2015 (inception of CHAMPS) through October 2023. Cases were excluded if death occurred before arrival at the health care facility, if the MITS or DeCoDe was not completed, and if clinical data were unavailable. Community deaths were included if there was a history of hospitalization and treatment prior to death.

### Clinical Care Guidelines 

The WHO and Ministries of Health in many countries have developed clinical care guidelines for common conditions among neonates to ensure standardized clinical care.^14^ We compared all documented clinical data to the clinical care guidelines outlined in the WHO Pocket Book of Hospital Care for Children.^14^ The Pocket Book guidelines (eTable 2 in Supplement 1) are tailored for health care professionals, adaptable to diverse global contexts, and focused on managing major causes of childhood mortality in low- and middle-income countries (LMICs).^14^ The Pocket Book has been widely adopted globally and is used at all CHAMPS sites.^17^

National guidelines for perinatal asphyxia and neonatal sepsis management at each of the 7 countries were reviewed to determine whether there were substantial differences in recommendations across countries.^18,19,20,21,22^ Given that the major diagnostic and therapeutic guidelines in these countries did not differ from the Pocket Book recommendations for these conditions, we focused on WHO guidelines in this analysis.

### Variables

Neonates who died from perinatal asphyxia or neonatal sepsis were identified by DeCoDe panels through consensus after review of integrated clinical, verbal autopsy, microbiological, and histopathologic data.^16^ In accordance with variables included in the Pocket Book recommendations, we extracted discrete clinical variables (ie, antibiotics administered) and narrative summaries pulled from clinical records of each case to identify clinical care provided to enrolled cases.

### Statistical Analysis

We used descriptive statistics for demographics of neonates who died and the proportion of neonates who died who received clinical care adherent to the Pocket Book guidelines. As the CHAMPS network has previously identified high rates of antimicrobial resistance to standard antibiotic therapy among neonates who died from neonatal sepsis, we calculated the proportion of cases of neonatal sepsis who received carbapenems (eg, meropenem) or cephalosporins (eg, ceftriaxone).^23^ We also performed subanalyses based on the country of residence and duration of hospital admission (<24 hours vs ≥24 hours) because we hypothesized that cases with longer hospital stays would be more likely to receive guideline-adherent care due to the increased time available for testing and treatment. Additionally, we hypothesized that neonates with low birth weight would be less likely to receive guideline-adherent care, as their symptoms may have been assumed to be associated with their prematurity and, therefore, inappropriate treatment.

We conducted mixed-effect univariable and multivariable logistic regression to identify factors associated with therapeutic administration of supplemental oxygen for perinatal asphyxia cases, bag-valve-mask (BVM) ventilation for perinatal asphyxia cases, and recommended antibiotics for neonatal sepsis, adjusting for factors identified in previous studies as having potential association with guideline adherence (ie, duration of hospitalization, age, sex, birth weight, and concordance of antemortem and postmortem diagnoses).^10,16^ Site was included as a random effect in each model. Variables were assessed for collinearity with generalized variance inflation factors. As we found no evidence of collinearity (ie, all generalized variance inflation factors ≤1.4), we included all candidate variables. The multivariable models included variables that met the statistical significance threshold of *P* ≤ .20 in the univariable comparisons. *P* < .05 was considered statistically significant in the main analyses, which were conducted using R, version 4.3.1 (R Project for Statistical Computing).

## Results

During the study period, CHAMPS enrolled 3479 neonates across the CHAMPS sites in 7 LMICs. Of these neonates, 1865 (53.6%) died from perinatal asphyxia or neonatal sepsis and 1194 (34.3%) underwent the full CHAMPS procedures to determine causes of death and had sufficient antemortem clinical data to be included in the present analyses (eFigure 1 in Supplement 1). A total of 476 neonates (39.9%) died from perinatal asphyxia, 562 (47.0%) died from neonatal sepsis, and 156 (13.1%) died from both conditions. Excluded cases were slightly younger than included cases and were more likely to have neonatal preterm birth complications and congenital birth defects as causes of death (eTable 3 in Supplement 1).

The included neonates (n = 1194) had a median (IQR) age at the time of death of 2 (1-6) days and comprised 692 males (58.0%), 501 females (42.0%), and 1 with indeterminate sex (0.1%) (Table 1). Those neonates had a median (IQR) gestational age of 36 (30-38) weeks and a median (IQR) birth weight of 2130 (1266-2988) g. Death occurred within 24 hours of admission for 429 neonates (36.6%). South Africa contributed the most cases (328 [27.5%]).

**Table 1.** Demographics of Neonates Who Died From Perinatal Asphyxia or Neonatal Sepsis in the CHAMPS Network

Characteristics	Neonates, No. (%)			
	Total (n = 1194)	Death from perinatal asphyxia (n = 476)	Death from neonatal sepsis (n = 562)	Death from both conditions (n = 156)
Age, median (IQR), d	2 (1-6)	1 (0-2)	5 (2-10)	3 (2-5)
<24 h	391 (32.7)	285 (59.9)	78 (13.9)	28 (17.9)
1-6 d	556 (46.6)	183 (38.4)	270 (48.0)	103 (66.0)
7-28 d	247 (20.7)	8 (1.7)	214 (38.1)	25 (16.0)
Sex				
Male	692 (58.0)	277 (58.2)	333 (59.3)	82 (52.6)
Female	501 (42.0)	198 (41.6)	229 (40.7)	74 (47.4)
Indeterminate	1 (0.1)	1 (0.2)	0	0
Gestational age, median (IQR), wk [n = 935]	36 (30-38)	38 (34-39)	31 (28-36)	38 (36-38)
Birth weight, median (IQR), g [n = 1106]	2130 (1266-2988)	2650 (1900-3100)	1405 (1000-2485)	2700 (2101-3200)
<1000	157 (14.2)	30 (6.5)	122 (23.8)	5 (3.7)
≥1000 to <1500	193 (17.5)	40 (8.7)	145 (28.3)	8 (6.0)
≥1500 to <2500	271 (24.5)	119 (25.9)	117 (22.9)	35 (26.1)
≥2500	485 (43.9)	271 (58.9)	128 (25.0)	86 (64.2)
Location of birth [n = 950]				
Hospital	724 (76.2)	302 (80.5)	330 (73.0)	92 (74.8)
Health center	148 (15.6)	48 (12.8)	83 (18.4)	17 (13.8)
Health post	12 (1.3)	8 (2.1)	3 (0.7)	1 (0.8)
Home: postdischarge deaths	48 (5.1)	12 (3.2)	25 (5.5)	11 (8.9)
On the way to health center	10 (1.1)	4 (1.1)	4 (0.9)	2 (1.6)
Other location	8 (0.8)	1 (0.3)	7 (1.5)	0
Age at hospital admission, d [n = 983]				
0	785 (79.9)	293 (84.7)	374 (75.7)	118 (82.5)
1	100 (10.2)	47 (13.6)	38 (7.7)	15 (10.5)
2	22 (2.2)	3 (0.9)	15 (3.0)	4 (2.8)
≥3	76 (7.7)	3 (0.9)	67 (13.6)	6 (4.2)
Congenital birth defects in causal chain or as other critical condition	108 (9.0)	38 (8.0)	63 (11.2)	7 (4.5)
Mode of delivery (n = 1193)				
Normal vaginal	774 (69.8)	293 (63.8)	366 (72.3)	115 (79.9)
Cesarean	334 (30.1)	166 (36.2)	140 (27.7)	28 (19.4)
Instrumentation (eg, forceps)	1 (0.1)	0	0	1 (0.7)
Location of death				
Community	17 (1.4)	10 (2.1)	6 (1.1)	1 (0.6)
Hospital	1177 (98.6)	466 (97.9)	556 (98.9)	155 (99.4)
Time of hospital death (n = 1171)				
Died in a facility <24 h	429 (36.6)	277 (59.8)	121 (21.8)	31 (20.1)
Died in a facility between 24 and <48 h	223 (19.0)	120 (25.9)	70 (12.6)	33 (21.4)
Died in a facility between 48 and <72 h	98 (8.4)	33 (7.1)	44 (7.9)	21 (13.6)
Died in a facility ≥72 h	421 (36.0)	33 (7.1)	319 (57.6)	69 (44.8)
Country site				
Bangladesh	192 (16.1)	117 (24.6)	44 (7.8)	31 (19.9)
Ethiopia	74 (6.2)	10 (2.1)	42 (7.5)	22 (14.1)
Kenya	113 (9.5)	72 (15.1)	41 (7.3)	0
Mali	70 (5.9)	21 (4.4)	46 (8.2)	3 (1.9)
Mozambique	251 (21.0)	121 (25.4)	108 (19.2)	22 (14.1)
Sierra Leone	166 (13.9)	80 (16.8)	35 (6.2)	51 (32.7)
South Africa	328 (27.5)	55 (11.6)	246 (43.8)	27 (17.3)

Abbreviation: CHAMPS, Child Health and Mortality Prevention Surveillance.

### Death From Perinatal Asphyxia

Among neonates who died, 632 (52.9%) had perinatal asphyxia anywhere in the causal chain of death. A combination of all recommended treatments was received by 4.4% of neonates (n = 28) who died with perinatal asphyxia. Airway suctioning was performed in 44.9% of cases (284), and BVM ventilation was performed in 43.2% of cases (273). Intravenous (IV) hydration was provided to 67.4% of cases (426), and chest compressions were performed in 65.2% of cases (412) (eTable 4 in Supplement 1). Administration of adrenaline occurred only in 12.2% of cases (77). Supplemental oxygen was administered to 85.4% of cases (540), primarily via nasal cannula (49.2% [311]), followed by continuous airway pressure (24.7% [156]). Only 8.1% of neonates (51) who died of perinatal asphyxia received none of the recommended treatments.

**Figure 1.**
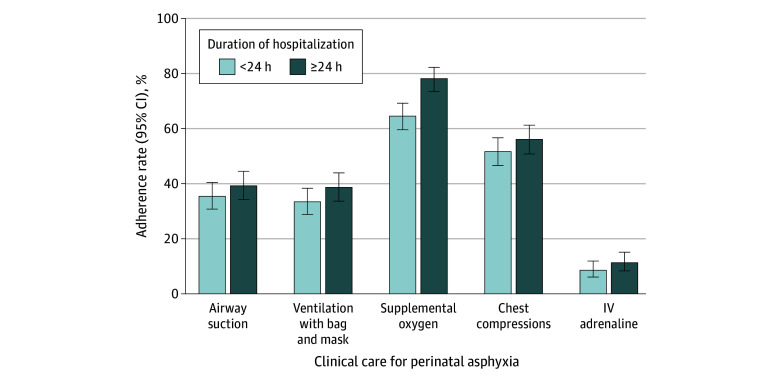
Adherence to World Health Organization Clinical Guidelines for Perinatal Asphyxia Stratified by Duration of Hospitalization (n = 617) Error bars represent 95% CIs. IV indicates intravenous.

For each of the diagnostics and therapeutics reviewed, neonates who were hospitalized for 24 hours or more before death had higher rates of guideline-adherent care compared with those hospitalized for less than 24 hours (Figure 1). However, this difference was significant only for supplemental oxygen (90.9% [95% CI, 87.0%-93.8%] vs 82.8% [95% CI, 78.0%-86.7%]; *P* < .001). Adherence to guidelines for perinatal asphyxia was highest in the South Africa sites (eg, BVM, 90.1%; 95% CI, 81.0%-95.3%) (eFigure 2 in Supplement 1).

Neonates for whom clinicians had documented perinatal asphyxia as an antemortem diagnosis were more likely to receive BVM ventilation (adjusted odds ratio [AOR] 2.00; 95% CI, 1.29-3.12) (Table 2). Otherwise, no variables were associated with the administration of BVM ventilation in multivariable analyses. Neonates who died after 24 hours of hospitalization, those who were older at death (ie, aged 7-28 days vs <24 hours), and those for whom clinicians had documented perinatal asphyxia before death had greater odds of receiving supplemental oxygen in univariable comparisons (eTable 5 in Supplement 1).

**Table 2.** Factors Associated With the Administration of Bag-Valve-Mask Ventilation Among Neonates Who Died From Perinatal Asphyxia^a^

Factor	Ventilation, No. (%)		OR (95% CI)	*P* value	AOR (95% CI)^b^	*P* value
	With BVM (n = 259)	Without BVM (n = 320)				
Time from admission to death						
Died in a facility <24 h	129 (49.8)	171 (53.4)	1 [Reference]	.38	1 [Reference]	.72
Died in a facility ≥24 h	130 (50.2)	149 (46.6)	1.16 (0.83-1.61)		0.90 (0.51-1.60)	
Age						
<24 h	136 (52.5)	161 (50.3)	1 [Reference]	.58	1 [Reference]	.45
1-6 d	113 (43.6)	141 (44.1)	0.95 (0.68-1.33)		0.84 (0.47-1.50)	
7-28 d	10 (3.9)	18 (5.6)	0.66 (0.28-1.45)		0.51 (0.18-1.44)	
Sex						
Female	119 (45.9)	134 (41.9)	1.18 (0.85-1.64)	.33	1.40 (0.95-2.06)	.09
Male	140 (54.1)	186 (58.1)	1 [Reference]		1 [Reference]	
Congenital anomalies						
Yes	15 (5.8)	28 (8.8)	1 [Reference]	.17	1 [Reference]	.32
No	244 (94.2)	292 (91.2)	1.56 (0.83-3.06)		1.40 (0.95-2.06)	
Concordant antemortem and postmortem diagnoses						
Yes	146 (56.4)	109 (34.1)	2.50 (1.79-3.51)	<.001	2.00 (1.29-3.12)	.002
No	113 (43.6)	211 (65.9)	1 [Reference]		1 [Reference]	
Birth weight, g						
<1000	22 (8.5)	12 (3.8)	2.20 (1.07-4.73)	.02	2.40 (0.88-6.51)	.36
≥1000 to <1500	15 (5.8)	33 (10.3)	0.55 (0.28-1.02)		0.93 (0.44-2.00)	
≥1500 to <2500	63 (24.3)	84 (26.2)	0.90 (0.61-1.33)		0.97 (0.61-1.56)	
≥2500	159 (61.4)	191 (59.7)	1 [Reference]		1 [Reference]	

Abbreviations: AOR, adjusted OR; BVM, bag-valve-mask ventilation; OR, odds ratio.

^a^ Complete case analysis excludes deaths with missing birth weight (n = 38), deaths occurring in the community or with missing duration of hospitalization (n = 14), and with indeterminate sex (n = 1).

^b^ All values are in mixed-effects model controlled for fixed effects for variables in column and random effect for site.

### Death From Neonatal Sepsis

There were 718 neonates who died from sepsis, and 28.8% of these neonates (n = 207) had blood cultures drawn before death (eTable 6 in Supplement 1). A combination of antemortem blood culture acquisition and administration of any recommended antibiotic treatment was observed in 24.1% of cases (173). Antibiotics (regardless of type) were administered to 86.8% of neonates (623). The administration of more than 1 antibiotic was common. Of these neonates, 61.0% (438) received the Pocket Book–recommended treatment of ampicillin or penicillin and gentamicin, and only 0.6% (4) received the recommended treatment of cloxacillin and gentamicin. Thirty-five percent of neonates (251) received ceftriaxone, and 17.5% (126) received meropenem. Seizures were observed in 26.9% of cases (193), among whom anticonvulsants were administered in 85.5% of cases (165).

**Figure 2.**
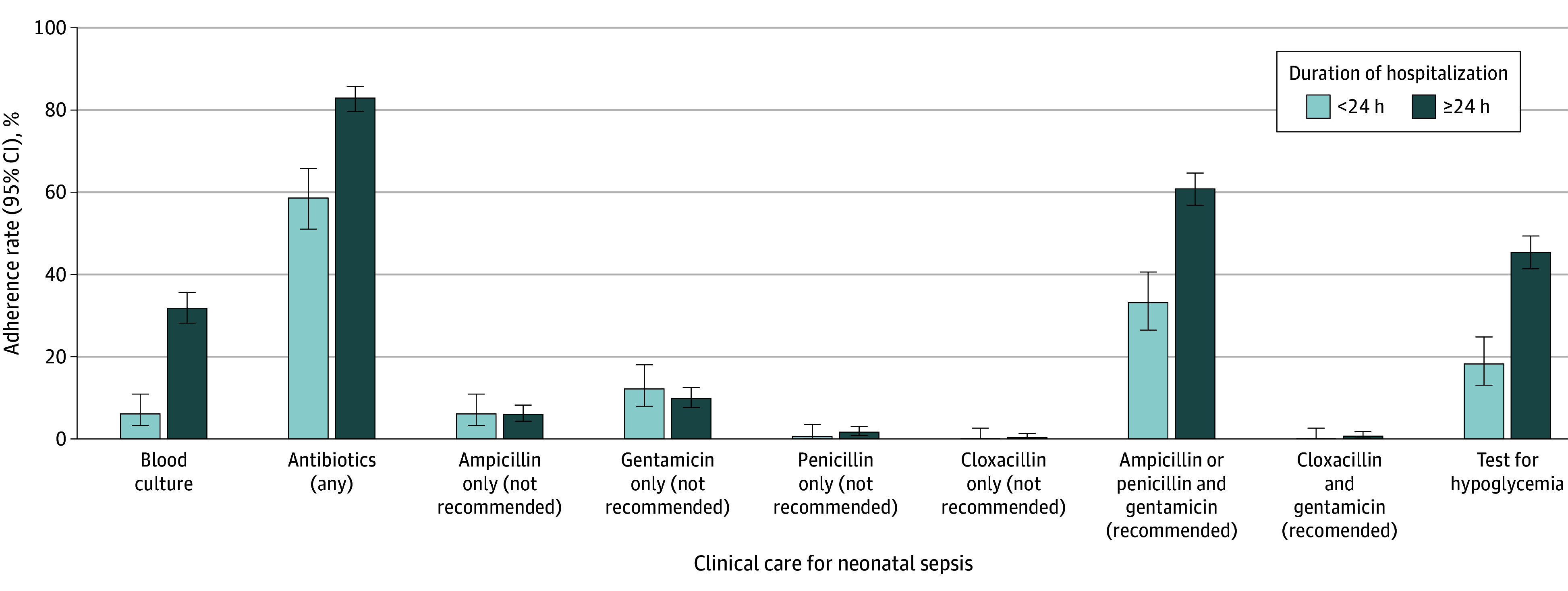
Adherence to World Health Organization Clinical Guidelines for Neonatal Sepsis Stratified by Duration of Hospitalization (N = 708) Error bars represent 95% CIs.

Neonates who were hospitalized for 24 hours or longer compared with those admitted for less than 24 hours had higher rates of having blood cultures drawn (35.3% [95% CI, 31.3%-39.4%] vs 7.2% [95% CI, 3.8%-12.9%]; *P* < .001) and receiving recommended antibiotics (67.6% [95% CI, 63.5%-71.5%] vs 39.5% [95% CI, 31.7%-47.7%]; *P* < .001) (Figure 2). The acquisition of blood cultures and the administration of recommended antibiotics were highest in the South Africa sites (eFigure 3 in Supplement 1). In univariable analyses, longer hospital admission (≥24 vs <24 hours: OR, 3.19 [95% CI, 2.17-4.72]; *P* < .001) and older age (1-6 days vs <24 hours: OR, 3.76 [95% CI, 2.39-6.01], *P* < .001; 7-28 days vs <24 hours: OR, 3.58 [95% CI, 2.20-5.89], *P* < .001) were associated with the administration of guideline-adherent antibiotics (Table 3). In multivariable analyses, older age at the time of death (1-6 days vs <24 hours: OR, 3.99 [95% CI, 2.08-7.66], *P* < .001; 7-28 days vs <24 hours: OR, 2.93 [95% CI, 1.48-5.77], *P* < .001) was associated with the administration of guideline-adherent antibiotics.

**Table 3.** Factors Associated With Guideline-Adherent Administration of Any Antibiotics Among Neonates With Neonatal Sepsis in the Causal Chain of Mortality^a^^,^^b^

Factor	Neonates, No. (%)		OR (95% CI)	*P* value	AOR (95% CI)^c^	*P* value
	With guideline-adherent antibiotics (n = 400)	Without guideline-adherent antibiotics (n = 240)				
Time from admission to death						
Died in a facility <24 h	56 (14.0)	82 (34.2)	1 [Reference]	<.001	1 [Reference]	.34
Died in a facility ≥24 h	344 (86.0)	158 (65.8)	3.19 (2.17-4.72)		1.32 (0.75-2.32)	
Age						
<24 h	38 (9.5)	67 (27.9)	1 [Reference]	<.001	1 [Reference]	<.001
1-6 d	222 (55.5)	104 (43.3)	3.76 (2.39-6.01)		3.99 (2.08-7.66)	
7-28 d	140 (35.0)	69 (28.7)	3.58 (2.20-5.89)		2.93 (1.48-5.77)	
Sex						
Female	157 (39.2)	108 (45.0)	0.79 (0.57-1.09)	.15	0.75 (0.52-1.09)	.13
Male	243 (60.8)	132 (55.0)	1 [Reference]		1 [Reference]	
Congenital anomalies						
Yes	34 (8.5)	29 (12.1)	1 [Reference]	.15	1 [Reference]	.64
No	366 (91.5)	211 (87.9)	1.48 (0.87-2.50)		1.17 (0.61-2.26)	
Concordant antemortem and postmortem diagnoses						
Yes	34 (8.5)	29 (12.1)	1.00 (0.52-1.98)	>.99	1.24 (0.58-2.62)	.56
No	366 (91.5)	211 (87.9)	1 [Reference]		1 [Reference]	
Birth weight, g						
<1000	88 (22.0)	39 (16.2)	2.07 (1.31-3.32)	.001	1.12 (0.62-2.02)	.17
≥1000 to <1500	109 (27.3)	44 (18.3)	2.27 (1.47-3.56)		1.79 (1.05-3.05)	
≥1500 to <2500	94 (23.5)	57 (23.8)	1.51 (0.99-2.32)		1.33 (0.82-2.16)	
≥2500	109 (27.3)	100 (41.7)	1 [Reference]		1 [Reference]	

Abbreviations: AOR, adjusted OR; OR, odds ratio.

^a^ Guideline-adherent antibiotics were ampicillin or penicillin and gentamicin or cloxacillin and gentamicin.

^b^ Complete case analysis excludes deaths with missing birth weight (n = 72) and deaths occurring in the community or with missing duration of hospitalization (n = 6).

^c^ All values are in mixed-effects model controlled for fixed effects for variables in column and random effect for site.

## Discussion

Adherence to the WHO Pocket Book guidelines for perinatal asphyxia and neonatal sepsis was suboptimal among neonates who died. Less than 5% of neonates who died from perinatal asphyxia received all recommended clinical care, approximately 60% of neonates who died from sepsis were given recommended antibiotics, and less than 25% of neonates who died from sepsis received both a blood culture and guideline-adherent antibiotics. For both perinatal asphyxia and neonatal sepsis, guideline adherence was higher among neonates who were hospitalized for 24 hours or longer. Significant variation in guideline adherence was observed across CHAMPS countries.

The finding that antemortem guideline adherence was suboptimal for deceased neonates is consistent with results of previous studies. In a prior study, supplemental oxygen was administered to 51% of infants and children who died from lower respiratory tract infections in the same settings, and IV fluids were administered to 16% of children who died from sepsis.^10^ Antibiotic use was common in neonates who died from sepsis. We observed that more than 1 in 3 neonates who died from sepsis received ceftriaxone and approximately 1 in 5 of these neonates received meropenem, which are not currently recommended by the WHO. This practice is likely informed by growing evidence of antimicrobial resistance among bacteria affecting neonates to commonly used antibiotics.^23^ Additional studies are warranted to better identify neonates at risk for highly resistant pathogens as a balance between adequate coverage, and avoiding the administration of overly broad-spectrum antibiotics is paramount. Such studies may inform adjustments to clinical care guidelines in the future.

We also observed country-level variation for the provision of guideline-adherent treatments. These variations in care adherence may stem from differences in the levels of resources available at each CHAMPS site, cost of clinical care, or availability of free health care, although the present study was not designed to elucidate reasons for variations. A study conducted in Nepal among neonates with sepsis demonstrated moderately high adherence (79%) to WHO guidelines.^13^ This adherence rate is higher than the rate observed in our population of neonates who died from sepsis. However, the study in Nepal did not confirm that neonates died of neonatal sepsis using diagnostics, and the authors acknowledged that cases may have died from other causes, which may have led to an overestimation of guideline adherence given the imprecision of postmortem diagnostics in that study. Therefore, the present study adds value by confirming the cause of death and adherence to care for neonates who died from neonatal sepsis and/or perinatal asphyxia.

Greater guideline adherence was observed among deceased neonates who were hospitalized for 24 hours or more compared with those who were admitted for less than 24 hours. This finding aligns with the results of prior studies in infants and children.^10^ Greater adherence for neonates with longer hospitalizations may be attributed to the additional time required to order laboratory tests, to obtain medications, and for clinicians to achieve diagnostic clarity to guide treatment. Similarly, for perinatal asphyxia, resuscitation may be performed at the time of birth, extending the length of hospitalization and the neonate’s life, although the neonate died later. Further research is necessary to understand the time of infection onset and the timing of resuscitation.

Given the well-documented phenomenon that a high proportion of neonatal deaths (up to 60%) occur within 24 hours of admission in resource-limited settings, there is an imperative to expedite diagnoses and administration of recommended clinical care.^24,25,26^ Interventions such as implementing formal triage processes, initiating treatment and stabilization before transferring patients to inpatient wards, and establishing dedicated pediatric emergency units have demonstrated effectiveness in inpatient mortality reduction.^27,28^

In the present study, less than 1 in 3 neonates had a blood culture drawn before their death. Several health facilities participating in this study, such as those in Ethiopia, do not have the capacity for routine cultures. Blood culture results are pivotal in determining appropriate antibiotic prescription. Increasing blood culture rates and decreasing result-turnaround times are critical.^29,30^ Prolonged waiting times for test results are commonly reported in LMICs and are often exacerbated by a lack of rapid communication of results.^31,32,33^ Effective and immediate communication of blood culture results to inform clinical treatment is critical.^34,35^ Moreover, there is a need to encourage timely presentation of ill neonates to health facilities, to reduce delays in the provision of high-quality health care, and to use enhanced infection control measures, as many neonates may have developed nosocomial infections.

There are several interventions that may improve guideline adherence among health care practitioners. These include sharing copies of recommendations, refresher trainings on guidelines, job aides, and feedback to clinicians on specific cases.^36^ Knowledge of clinical care guidelines is crucial because it enables clinicians to make evidence-based decisions efficiently by offering guidance on diagnostics, procedures, and medications. By promoting interventions of proven benefit and enhancing consistency of care, clinical guidelines promote the provision of higher-quality clinical care.^37^ A systematic review of 59 studies that evaluated clinical care guidelines reported that clinical care significantly improved in all but 4 of the studies after the introduction of clinical care guidelines in health care facilities.^38^ Consequently, additional efforts to educate health care practitioners and encourage adherence to clinical care recommendations are needed in regions with high childhood mortality to improve health outcomes for neonates. Moreover, studies assessing health system challenges, such as availability of diagnostics and therapeutics and adequate staffing, are warranted.

### Limitations

There are several limitations to this study. First, CHAMPS does not collect data on neonates who survive, which limited our ability to measure the association of adherence to recommendations with reduced mortality. Future studies should assess the potential differences in guideline adherence between neonates who survive and those who die. Second, this study relied on clinical records documented by clinicians and nurses. Consequently, there is a possibility that some aspects of clinical care were provided but not documented, potentially leading to overestimations of shortcomings in clinical care. This study did not evaluate doses, duration, or changes in medications. It only considered whether a medication was administered or not, thus making it difficult to conclude whether WHO dosing and duration guidelines were followed. Although CHAMPS includes sites in 7 LMICs in sub-Saharan Africa and South Asia, the findings may not be representative of guidelines adherence in other regions or within the same regions with differing health care facility resources. Moreover, perinatal asphyxia management largely relies on initial resuscitation. However, the timing of interventions was not always documented; thus, we were unable to assess if interventions were administered in a timely fashion. Third, it was not possible to determine why recommended diagnostics and therapeutics were not provided, although such a study is currently being conducted in the CHAMPS network.

## Conclusions

The finding of low adherence to WHO clinical care guidelines underscores the critical need to increase adherence in regions with high rates of neonatal mortality. Additionally, insights from this study highlight the need to evaluate barriers to adherence and may inform strategies for strengthening health systems to support compliance with clinical care guidelines.

## Notes

Supplement 1. **eFigure 1.** Included Cases of Perinatal Asphyxia or Neonatal Sepsis Deaths, 2015-2023 **eFigure 2**. Adherence to WHO Recommendations for Birth Asphyxia Determined by Postmortem Diagnosis by Site (N=617) **eFigure 3**. Adherence to WHO Recommendations for Neonatal Sepsis Determined by Postmortem Diagnosis by Site (N=708) **eTable 1.** Healthcare Facilities at Each CHAMPS Site **eTable 2.** WHO Pocket Book of Hospital Care for Children Perinatal Asphyxia and Neonatal Sepsis Guidelines **eTable 3.** Comparison of Deceased Neonates With MITS and DeCoDe in the CHAMPS Network Included and Excluded in the Analysis of Clinical Care Recommendation Adherence **eTable 4.** Adherence to WHO Pocket Book of Hospital Care for Children Recommendations for Perinatal Asphyxia Determined by Postmortem Diagnosis (N=632) **eTable 5.** Factors Associated With Any Supplemental Oxygen for Birth Asphyxia Among Neonates Who Had Birth Asphyxia in the Causal Chain of Mortality (N=579) **eTable 6.** Adherence to WHO Pocket Book of Hospital Care for Children Recommendations for Neonatal Sepsis Determined by Postmortem Diagnosis (N=718)

Supplement 2.
Nonauthor Collaborators

Supplement 3.
Data Sharing Statement
