# Supplementary material for: Adherence to Perinatal Asphyxia or Sepsis Management Guidelines in Low- and Middle-Income Countries
Source: JAMA Netw Open. 2025 May 16;8(5):e2510790. doi: 10.1001/jamanetworkopen.2025.10790 (PMC12084843; doi:10.1001/jamanetworkopen.2025.10790)
Supplement: Supplement 1. — eFigure 1. Included Cases of Perinatal Asphyxia or Neonatal Sepsis Deaths, 2015-2023 eFigure 2. Adherence to WHO Recommendations for Birth Asphyxia Determined by Postmortem Diagnosis by Site (N=617) eFigure 3. Adherence to WHO Recommendations for Neonatal Sepsis Determined by Postmortem Diagnosis by Site (N=708) eTable 1. Healthcare Facilities at Each CHAMPS Site eTable 2. WHO Pocket Book of Hospital Care for Children Perinatal Asphyxia and Neonatal Sepsis Guidelines eTable 3. Comparison of Deceased Neonates With MITS and DeCoDe in the CHAMPS Network Included and Excluded in the Analysis of Clinical Care Recommendation Adherence eTable 4. Adherence to WHO Pocket Book of Hospital Care for Children Recommendations for Perinatal Asphyxia Determined by Postmortem Diagnosis (N=632) eTable 5. Factors Associated With Any Supplemental Oxygen for Birth Asphyxia Among Neonates Who Had Birth Asphyxia in the Causal Chain of Mortality (N=579) eTable 6. Adherence to WHO Pocket Book of Hospital Care for Children Recommendations for Neonatal Sepsis Determined by Postmortem Diagnosis (N=718) [file jamanetwopen-e2510790-s001.pdf]

Supplemental Online Content

Rahman A, Ray M, Madewell ZJ, et al; Child Health and Mortality Prevention Surveillance (CHAMPS) Network. Adherence to perinatal asphyxia or sepsis management guidelines in low- and middle-income countries. *JAMA Netw Open*. 2025;8(5):e2510790. doi:10.1001/jamanetworkopen.2025.10790

eFigure 1. Included Cases of Perinatal Asphyxia or Neonatal Sepsis Deaths, 2015-2023
eFigure 2. Adherence to WHO Recommendations for Birth Asphyxia Determined by Postmortem Diagnosis by Site (N=617)
eFigure 3. Adherence to WHO Recommendations for Neonatal Sepsis Determined by Postmortem Diagnosis by Site (N=708)
eTable 1. Healthcare Facilities at Each CHAMPS Site
eTable 2. WHO Pocket Book of Hospital Care for Children Perinatal Asphyxia and Neonatal Sepsis Guidelines
eTable 3. Comparison of Deceased Neonates With MITS and DeCoDe in the CHAMPS Network Included and Excluded in the Analysis of Clinical Care Recommendation Adherence
eTable 4. Adherence to WHO Pocket Book of Hospital Care for Children Recommendations for Perinatal Asphyxia Determined by Postmortem Diagnosis (N=632)
eTable 5. Factors Associated With Any Supplemental Oxygen for Birth Asphyxia Among Neonates Who Had Birth Asphyxia in the Causal Chain of Mortality (N=579)
eTable 6. Adherence to WHO Pocket Book of Hospital Care for Children Recommendations for Neonatal Sepsis Determined by Postmortem Diagnosis (N=718)

This supplemental material has been provided by the authors to give readers additional information about their work.

© 2025 Rahman A et al. *JAMA Network Open*

eFigure 1. Included cases of perinatal asphyxia or neonatal sepsis deaths, 2015-2023

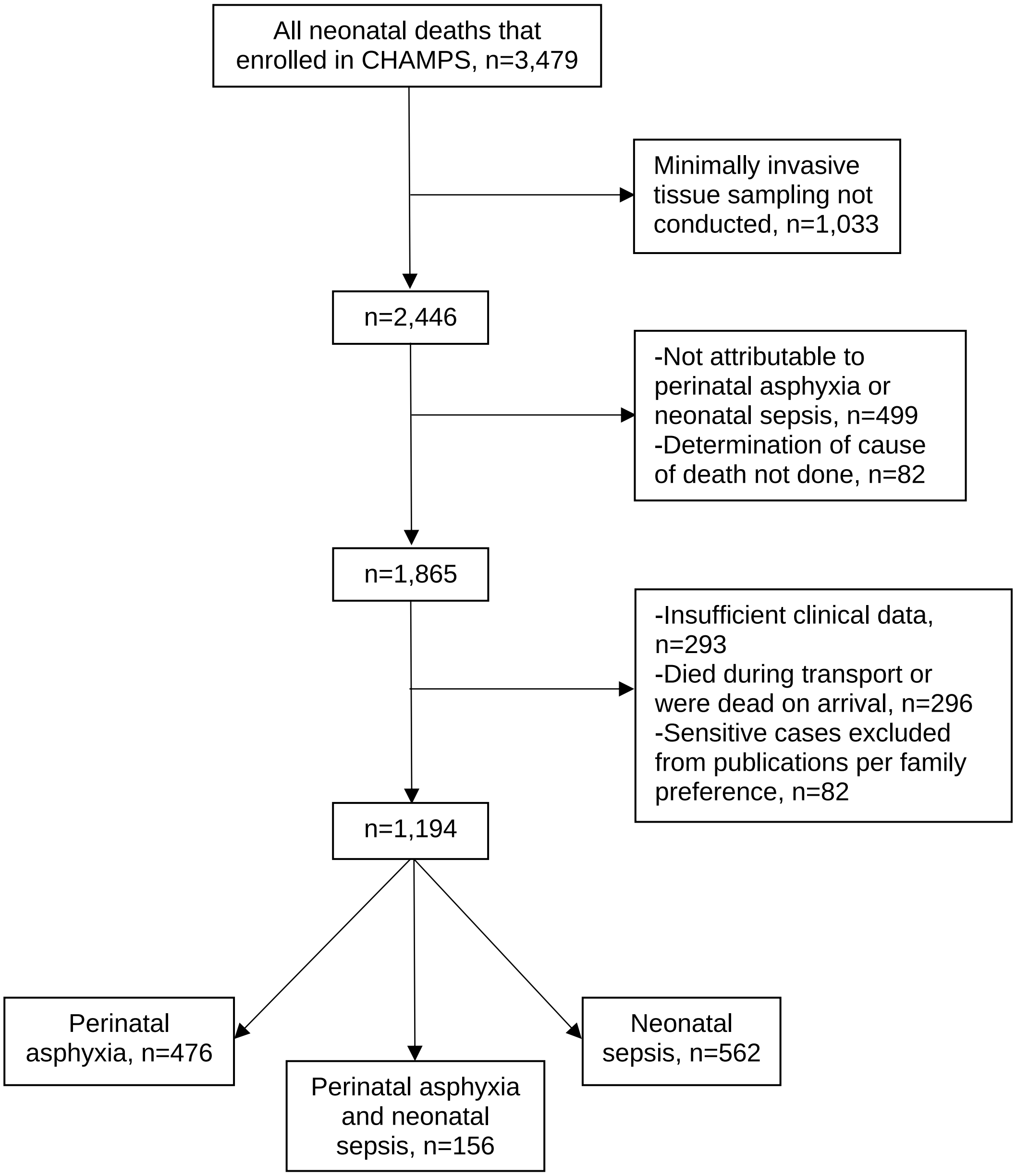

© 2025 Rahman A et al. *JAMA Network Open*

eFigure 2. Adherence to WHO recommendations for birth asphyxia determined by postmortem diagnosis by site* (N=617)

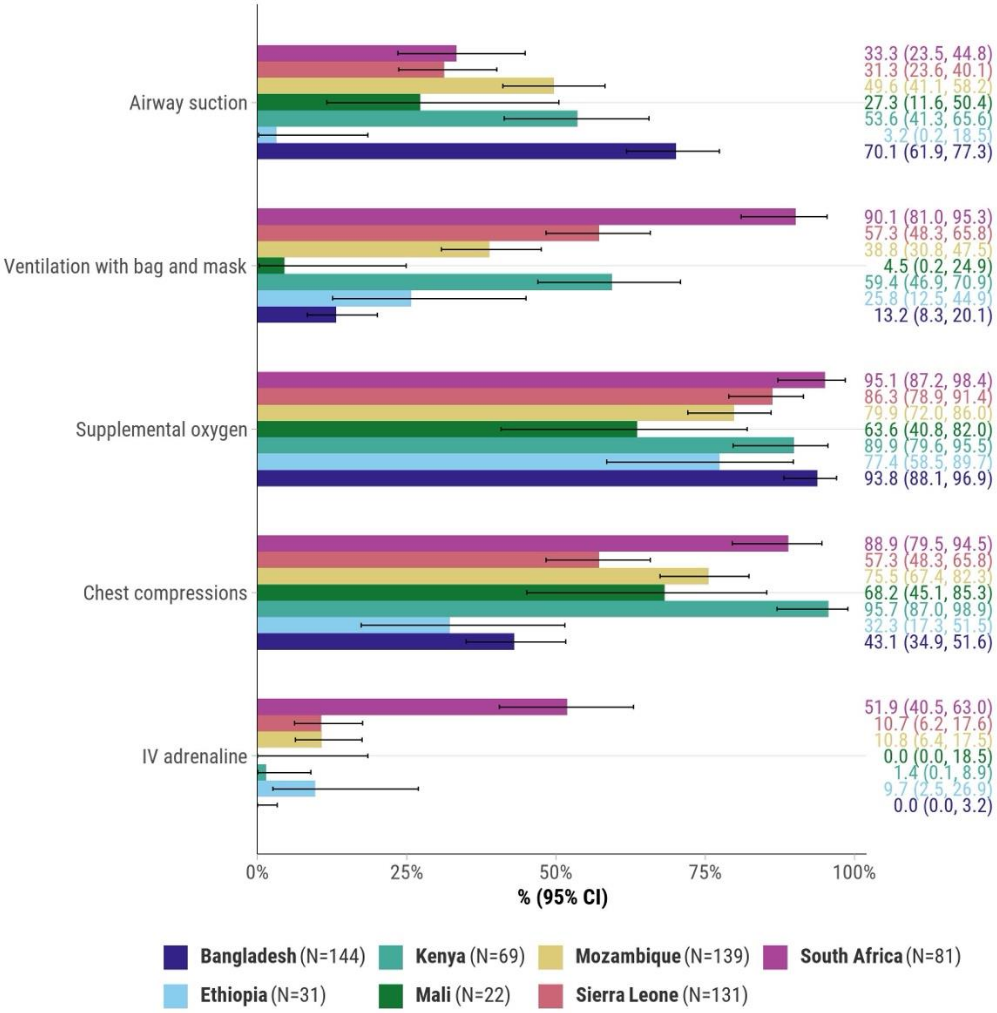

*Error bars represent 95% confidence intervals.

© 2025 Rahman A et al. *JAMA Network Open*

eFigure 3. Adherence to WHO recommendations for neonatal sepsis determined by postmortem diagnosis by site (N=708)

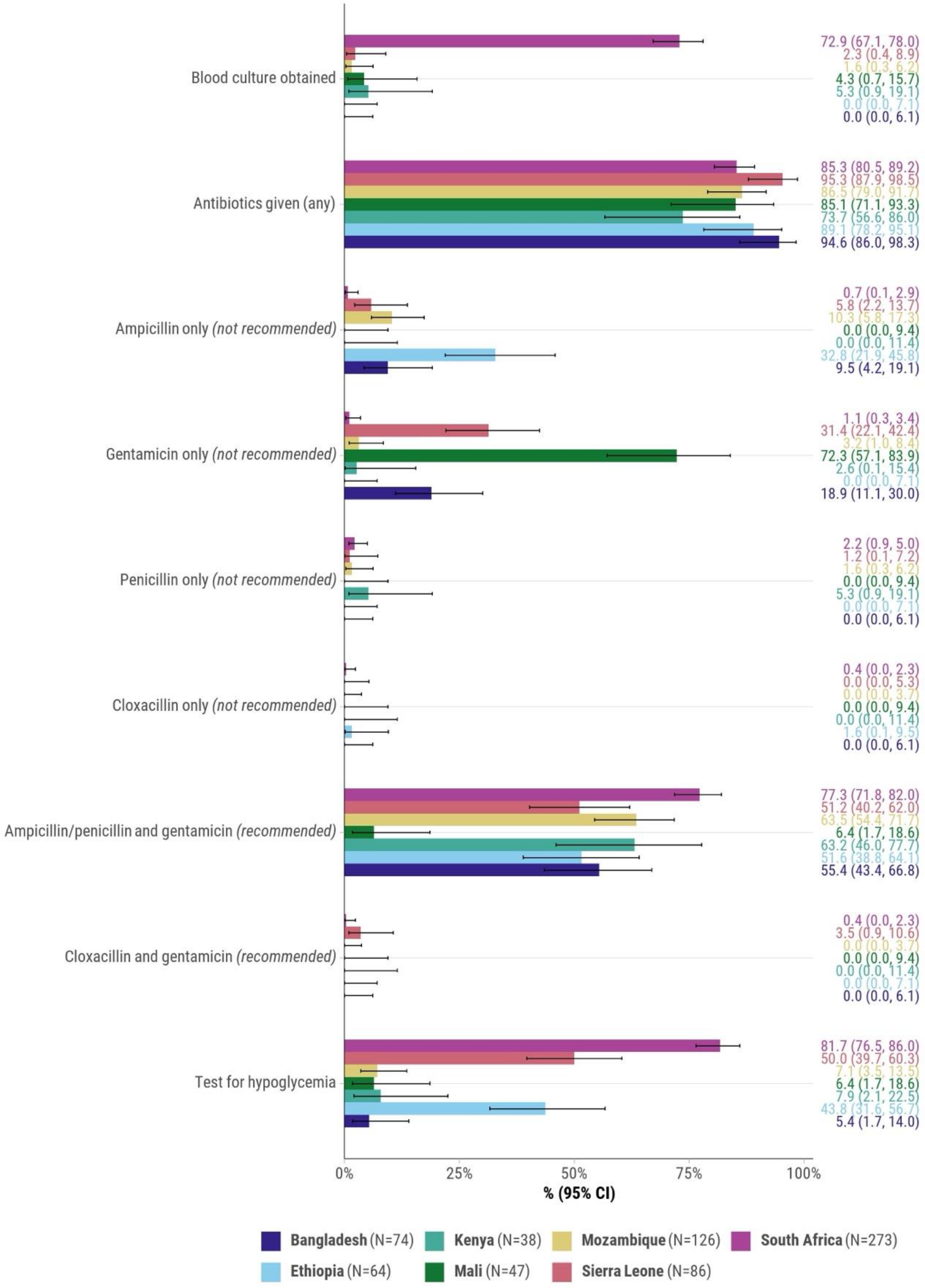

*Error bars represent 95% confidence intervals.

© 2025 Rahman A et al. *JAMA Network Open*

eTable 1. Healthcare facilities at each CHAMPS site

CHAMPS Site	Hospital(s)	Type of Hospital
Bangladesh	-Baliakandi Upazila Health Complex<br>-Faridpur Medical College Hospital<br>-Zahid Memorial Children Hospital	-Urban referral hospital<br>-Urban referral hospital<br>-Peri-urban hospital
Ethiopia	Hiwot Fana Comprehensive Specialized Hospital	Urban referral hospital
Kenya	-Jaramogi Oginga Odinga Teaching & Referral Hospital (Kisumu)<br>-Siaya County Referral Hospital (Siaya)	-Urban referral hospital<br>-Rural referral hospital
Mali	Hôpital Gabriel Touré	Urban referral hospital
Mozambique	-Manhiça District Hospital<br>-Central Hospital of Quelimane	-Urban district hospital<br>-Rural regional hospital
Sierra Leone	Makeni Regional Hospital	Rural regional hospital
South Africa	Chris Hani Baragwanath Hospital	Urban referral hospital

© 2025 Rahman A et al. *JAMA Network Open*

eTable 2. WHO Pocket Book of Hospital Care for Children perinatal asphyxia and neonatal sepsis guidelines*

**Birth Asphyxia**
• Assess breathing and check heart rate with a stethoscope<br>• Airway suction if infant had meconium-stained liquor or the mouth or nose is full of secretions<br>• Positive pressure ventilation with mask and self-inflating bag if infant is not breathing or gasping<br>• Provide high concentration oxygen if heart rate is <60/min or between 60 – 100/min<br>• Provide chest compressions if heart rate is <60/min<br>• Provide other ventilatory support if heart rate is <60/min<br>• Provide IV adrenaline if heart rate is <60/min
**Neonatal Sepsis**
• Empirical antibiotics should be given to children with suspected neonatal sepsis<br>• When possible, perform a lumbar puncture and obtain blood cultures before starting antibiotics<br>• For newborns with any signs of serious bacterial infection or sepsis, give ampicillin (or penicillin) and gentamicin as a first-line antibiotic treatment<br>• If at greater risk of staphylococcus infection (extensive skin pustules, abscess or omphalitis in addition to signs of sepsis), give IV cloxacillin and gentamicin<br>• If the infant is drowsy or unconscious, ensure that hypoglycemia is not present; if it is, give 2 ml/kg 10% glucose IV<br>• Treat convulsions with phenobarbital<br>• If the child is from a malarious area and has fever, take a blood film to check for malaria. If confirmed, treat with artesunate or quinine.

*World Health Organization. *Pocket book of hospital care for children: guidelines for the management of common childhood illnesses.* World Health Organization; 2013.

© 2025 Rahman A et al. *JAMA Network Open*

eTable 3. Comparison of deceased neonates with MITS and DeCoDe in the CHAMPS network included and excluded in the analysis of clinical care recommendation adherence

	**Included in Analyses (N=1,194), n (%)**	**Excluded from Analyses (N=947), n (%)**	***P* value***
**Patient Age, days (median, [IQR])**	2 [1, 6]	1 [0, 3]	<0.001
<24 hours	391 (32.7)	440 (46.5)	
1 – 6 days	556 (46.6)	383 (40.4)	
7 – 28 days	247 (20.7)	124 (13.1)	
**Sex**			0.123
Male	692 (58.0)	544 (57.4)	
Female	501 (42.0)	401 (42.3)	
Indeterminate	1 (0.1)	2 (0.3)	
**Causes of Death**			
Neonatal preterm birth complications	409 (34.1)	516 (54.4)	<0.001
Other neonatal disorders	134 (9.8)	65 (6.9)	0.282
Congenital birth defects	79 (6.2)	102 (10.8)	<0.001
Neonatal aspiration syndromes	44 (4.3)	71 (7.5)	0.001
Neonatal encephalopathy	71 (5.0)	31 (3.3)	0.608

*calculated by Chi-square test

© 2025 Rahman A et al. *JAMA Network Open*

eTable 4. Adherence to WHO Pocket Book of Hospital Care for Children recommendations for perinatal asphyxia determined by postmortem diagnosis (N=632)

**Recommendation**	n (%)
Airway Suction	284 (44.9)
IV Hydration	426 (67.4)
**Resuscitation**	
Ventilation with bag-valve-mask	273 (43.2)
Chest compressions	412 (65.2)
Adrenaline	77 (12.2)
**Supplemental Oxygen***	
Any supplemental oxygen	540 (85.4)
Blow-by-air	44 (7.0)
Nasal cannula	311 (49.2)
Continuous Positive Air Pressure (CPAP)	156 (24.7)
Bilevel Positive Air Pressure (BiPAP)	1 (0.2)
**All Recommended Treatments** (airway suction, ventilation with bag-valve mask, chest compressions, adrenaline)	28 (4.4)
**No Recommended Treatments**	51 (8.1)

*WHO guidelines state “Positive pressure ventilation should be initiated with air for infants with gestation > 32 weeks. For very preterm infants, it is preferable to start with 30% oxygen if possible.”

© 2025 Rahman A et al. *JAMA Network Open*

eTable 5. Factors associated with any supplemental oxygen for birth asphyxia among neonates who had birth asphyxia in the causal chain of mortality (N=579)[a]

	**Any supplemental oxygen N=501, n (%)**	**No supplemental oxygen N=78 n (%)**	**Odds Ratio (95% CI)**	***P* value**	**Adjusted Odds[b] Ratio (95% CI)**	***P* value**
**Time from admission to death**				0.009		0.19
Died in a facility <24 hours	249 (49.7)	51 (65.4)	*Referent*		*Referent*	
Died in a facility ≥24 hours	252 (50.3)	27 (34.6)	1.91 (1.17, 3.18)		1.64 (0.77, 3.49)	
**Patient Age, days**				0.03		0.34
<24 hours	248 (49.5)	49 (62.8)	*Referent*		*Referent*	
1-6 days	226 (45.1)	28 (35.9)	1.59 (0.98, 2.65)		1.07 (0.51, 2.28)	
7-28 days	27 (5.4)	1 (1.3)	5.33 (1.10, 96.27)		4.89 (0.58, 41.19)	
**Sex**				0.15		0.16
Female	213 (42.5)	40 (51.3)	0.70 (0.43, 1.13)		0.70 (0.43, 1.15)	
Male	288 (57.5)	38 (48.7)	*Referent*		*Referent*	
**Congenital anomalies**				0.16		0.27
Yes	34 (6.8)	9 (11.5)	*Referent*		*Referent*	
No	467 (93.2)	69 (88.5)	1.79 (0.78, 3.75)		1.62 (0.69, 3.81)	
**Concordant antemortem and postmortem diagnoses**				0.01		0.001
Yes	231 (46.1)	24 (30.8)	1.92 (1.17, 3.26)		2.62 (1.46, 4.70)	
No	270 (53.9)	54 (69.2)	*Referent*		*Referent*	
**Birthweight**				0.42		0.77
<1,000 grams	32 (6.4)	2 (2.6)	2.67 (0.77, 16.78)		1.86 (0.40, 8.65)	
≥1,000 to <1,500 grams	40 (8.0)	8 (10.3)	0.83 (0.39, 2.01)		0.77 (0.32, 1.85)	
≥1500 grams to <2,500 grams	129 (25.7)	18 (23.1)	1.19 (0.68, 2.18)		1.04 (0.56, 1.93)	
≥2,500 grams	300 (59.9)	50 (64.1)	*Referent*		*Referent*	

[a] Complete case analysis excludes deaths missing birthweight (N=38), deaths that occurred in the community or were missing duration of hospitalization (n=14), and with indeterminate sex (n=1).
[b] All values are in mixed effects model controlled for fixed effects for variables in column and random effect for site.

© 2025 Rahman A et al. *JAMA Network Open*

eTable 6. Adherence to WHO Pocket Book of Hospital Care for Children recommendations for neonatal sepsis determined by postmortem diagnosis (N=718)

**Recommendation**	**n (%)**
**Blood Culture**	207 (28.8)
**Antibiotics**	
Any antibiotic	623 (86.8)
Ampicillin only *(not recommended)*	48 (6.7)
Gentamicin only *(not recommended)*	84 (11.7)
Cloxacillin only *(not recommended)*	2 (0.3)
Ampicillin/penicillin AND gentamicin (*recommended*)	438 (61.0)
Cloxacillin AND gentamicin (*recommended*)	4 (0.6)
**Seizures**	
Seizures	193 (26.9)
Anticonvulsant (*if neonate had seizures*)	165 (85.5)
**Blood Culture and Recommended Antibiotics** (ampicillin/penicillin and gentamicin OR cloxacillin and gentamicin)	173 (24.1)
**No Recommended Treatments**	212 (29.5)

© 2025 Rahman A et al. *JAMA Network Open*
