# Supplementary material for: Adherence to Perinatal Asphyxia or Sepsis Management Guidelines in Low- and Middle-Income Countries
Source: JAMA Netw Open. 2025 May 16;8(5):e2510790. doi: 10.1001/jamanetworkopen.2025.10790 (PMC12084843; doi:10.1001/jamanetworkopen.2025.10790)
Supplement: Supplement 2. — Nonauthor Collaborators [file jamanetwopen-e2510790-s002.pdf]

Supplemental Online Content: Nonauthor Collaborators
*First name, last name, and suffix (if applicable) are required and will appear in PubMed.

***Group Name(s): Child Health and Mortality Prevention Surveillance Network**

***First Name and Middle Initial(s)**	***Last Name**	***Suffix (eg, Jr, III)**	Academic Degrees	Institution	Location (city, state/province, country)	Role or Contribution, eg, chair, principal investigator	Group (if more than 1 Group listed in the byline) and/or Subgroup (eg, Steering Committee)
Sanwarul	Bari		MD	Maternal and Child Health Division, Inter	Dhaka, Bangladesh	Data collection	
Shahana	Parveen		MSS	International Centre for Diarrhoeal Disea	Dhaka, Bangladesh	Data collection	
Mohammed	Kamal		PhD	Bangabandhu Sheikh Mujib Medical Univ	Dhaka, Bangladesh	Data collection	
A.S.M. Nawshad Uddin	Ahmed		FCPS	Bangladesh Institute of Child Health at D	Dhaka, Bangladesh	Data collection	
Mahbubul	Hoque		FCPS	Bangladesh Institute of Child Health at D	Dhaka, Bangladesh	Data collection	
Saria	Tasnim		FCPS	Dhaka Community Medical College and H	Dhaka, Bangladesh	Data collection	
Ferdousi	Islam		FCPS	Popular Medical College and Hospital in I	Dhaka, Bangladesh	Data collection	
Farida	Ariuman		FCPS	National Institute of Cancer Research and	Dhaka, Bangladesh	Data collection	
Mohammad M	Rahman		MD	Bangabandhu Sheikh Mujib Medical Univ	Dhaka, Bangladesh	Data collection	
Ferdousi	Begum		MD	Bangabandhu Sheikh Mujib Medical Univ	Dhaka, Bangladesh	Data collection	
K.	Zaman		PhD	International Centre for Diarrhoeal Disea	Dhaka, Bangladesh	Data collection	
Mustafizur	Rahman		PhD	International Centre for Diarrhoeal Disea	Dhaka, Bangladesh	Data collection	
Dilruba	Ahmed		PhD	International Centre for Diarrhoeal Disea	Dhaka, Bangladesh	Data collection	
Meerjady S	Flora		PhD	Institute of Epidemiology, Disease Contro	Dhaka, Bangladesh	Data collection	
Tahmina	Shirin		PhD	Institute of Epidemiology, Disease Contro	Dhaka, Bangladesh	Data collection	
Mahbubur	Rahman		MPH	Institute of Epidemiology, Disease Contro	Dhaka, Bangladesh	Data collection	
Joseph	Oundo		PhD	1) London School of Hygiene & Tropical M	Harar, Ethiopia	Data collection	
Alexander M	Ibrahim		MD	College of Health and Medical Sciences a	Harar, Ethiopia	Data collection	
Fikremelekot	Temesgen		MD	Addis Ababa University	Addis Ababa, Ethiopia	Data collection	
Tadesse	Gure		MD	College of Health and Medical Sciences a	Harar, Ethiopia	Data collection	
Addisu	Alemu		MD	College of Health and Medical Sciences a	Harar, Ethiopia	Data collection	
Melisachew M	Yeshi		MD	Ayder Specialized Comprehensive Hospit	Harar, Ethiopia	Data collection	
Mahlet A	Gizaw		MD	St. Paul’s Hospital Millennium Medical Co	Addis Ababa, Ethiopia	Data collection	
Stian MS	Orlien		PhD	London School of Hygiene & Tropical Me	London, United Kingdom	Data collection	
Solomon	Ali		PhD	National Data Management Centre at the	Addis Ababa, Ethiopia	Data collection	
Peter	Otieno		MA	The University of Nairobi, Kenya	Nairobi, Kenya	Data collection	
Peter N	Onyango		MA	The University of Nairobi, Kenya	Nairobi, Kenya	Data collection	
Janet	Agaya		MPH	Maseno University, Kenya	Kisumu, Kenya	Data collection	

Supplemental Online Content: Nonauthor Collaborators
*First name, last name, and suffix (if applicable) are required and will appear in PubMed.

***First Name and Middle Initial(s)**	***Last Name**	***Suffix (eg, Jr, III)**	Academic Degrees	Institution	Location (city, state/province, country)	Role or Contribution, eg, chair, principal investigator	Group (if more than 1 Group listed in the byline) and/or Subgroup (eg, Steering Committee)
Richard	Oliech		Diploma in	Kenya Polytechnic	Kisumu, Kenya	Data collection	
Joyce A	Were		MSc	University of Nairobi, Kenya	Nairobi, Kenya	Data collection	
Dickson	Gethi		BSc	University of Nairobi, Kenya	Nairobi, Kenya	Data collection	
George	Aol		MA	Great Lakes University of Kisumu, Ke	Kisumu, Kenya	Data collection	
Thomas	Misore		MA	The University of Nairobi, Kenya	Nairobi, Kenya	Data collection	
Harun	Owuor		MSc	Jaramogi Oginga Odinga University o	Kisumu, Kenya	Data collection	
Christopher	Muga		BSc	Jaramogi Oginga Odinga University o	Kisumu, Kenya	Data collection	
Bernard	Oluoch		Diploma in	Kenya Medical Training Institute, Nye	Nyeri, Kenya	Data collection	
Christine	Ochola		Diploma in	Kenya Medical Training Institute, Nye	Nyeri, Kenya	Data collection	
Sharon M	Tennant		PhD	University of Maryland School of Medicir	Baltimore, MD, United Stat	Data collection	
Carol L	Greene		MD	University of Maryland School of Medicir	Baltimore, MD, United Stat	Data collection	
Ashka	Mehta		MPH	Department of Pediatrics, Center for	Baltimore, MD, United Stat	Data collection	
J. Kristie	Johnson		PhD	University of Maryland School of Med	Baltimore, MD, United Stat	Data collection	
Brigitte	Gaume		PhD	Center for Vaccine Development and	Baltimore, MD, United Stat	Data collection	
Rima	Koka		MD	University of Maryland School of Med	Baltimore, MD, United Stat	Data collection	
Karen D	Fairchild		MD	University of Virginia	Charlottesville, VA, United	Data collection	
Diakaridia	Kone		MD	CSRef Commune I, Bamako, Mali	Bamako, Mali	Data collection	
Diakaridia	Sidibe		MD	Centre pour le Développement des V	Bamako, Mali	Data collection	
Doh	Sanogo		MD	Epidemiology Department CVD-Mali,	Bamako, Mali	Data collection	
Uma U	Onwuchekwa		MSc	Bioinformatics department, CVD-Mal	Bamako, Mali	Data collection	
Nana	Kourouma		MD, PHD	CVD-Mali, HGT, Bamako, Mali	Bamako, Mali	Data collection	
Seydou	Sissoko		MD	CVD-Mali, HGT, Bamako, Mali	Bamako, Mali	Data collection	
Cheick B	Traore		MD	CHU POINT G, Bamako Mali	Bamako, Mali	Data collection	
Jane	Juma		Ms, HND ir	CVD-Mali, HGT, Bamako, Mali	Bamako, Mali	Data collection	
Kounandji	Diarra		MSc	CNAM/CVD-Mali, Bamako, Mali	Bamako, Mali	Data collection	
Awa	Traore		MSc	CNAM/CVD-Mali, Bamako, Mali	Bamako, Mali	Data collection	
Tiéman	Diarra		PhD, Profe	Point-Sud, Bamako, Mali	Bamako, Mali	Data collection	
Kiranpreet	Chawla		MD	Department of Obstetrics, Gynecolog	Baltimore, MD, United Stat	Data collection	

Supplemental Online Content: Nonauthor Collaborators

*First name, last name, and suffix (if applicable) are required and will appear in PubMed.

***First Name and Middle Initial(s)**	***Last Name**	***Suffix (eg, Jr, III)**	Academic Degrees	Institution	Location (city, state/province, country)	Role or Contribution, eg, chair, principal investigator	Group (if more than 1 Group listed in the byline) and/or Subgroup (eg, Steering Committee)
Khátia	Munguambe			Centro de Investigacao em Saude de Manhica Eduardo Mondlane University, Faculty of Medicine, Community Health Department, Maputo, Mozambique	Maputo, Mozambique	Data collection	
Ariel	Nhacolo			Centro de Investigação em Saúde de	Maputo, Mozambique	Data collection	
Maria	Maixenchs			IS Global Hospital Clinic--Universitat d	Barcelona, Spain	Data collection	
Kyu H	Lee		PhD	Emory Global Health Institute, Emory Un	Atlanta, GA, United States	Data collection	
Shailesh	Nair		MPH	Public Health Informatics Institute, The T	Atlanta, GA, United States	Data collection	
Lucy	Liu		MBA	Public Health Informatics Institute at the	Atlanta, GA, United States	Data collection	
Courtney	Bursuc		MPH	Emory Global Health Institute, Emory	Atlanta, GA, United States	Data collection	
Kristin	LaHatte		MA	Emory Global Health Institute, Emory	Atlanta, GA, United States	Data collection	
Sarah	Raymer		BA	Emory Global Health Institute, Emory	Atlanta, GA, United States	Data collection	
John	Blevins		ThD	Emory Global Health Institute, Emory	Atlanta, GA, United States	Data collection	
Solveig	Argeseanu		PhD	Emory Global Health Institute, Emory	Atlanta, GA, United States	Data collection	
Kurt	Vyas		PhD	Emory Global Health Institute, Emory	Atlanta, GA, United States	Data collection	
Manu	Bhandari		MPH	Emory Global Health Institute, Emory	Atlanta, GA, United States	Data collection	
Mischka	Garel		MPH	Emory Global Health Institute, Emory Un	Atlanta, GA, United States	Data collection	
Navit T	Salzberg		MPH	Emory Global Health Institute, Emory Un	Atlanta, GA, United States	Data collection	
Jeffrey P	Koplan		MD	Emory Global Health Institute, Emory Un	Atlanta, GA, United States	Data collection	
Jana	Ritter		DVM	Infectious Diseases Pathology Branch, Di	Atlanta, GA, United States	Data collection	
Tais	Wilson		DVM	Centers for Disease Control and Prev	Atlanta, GA, United States	Data collection	
Jonas	Winchell		PhD	Respiratory Diseases Branch, Division of	Atlanta, GA, United States	Data collection	
Jakob	Witherbee		BS	Centers for Disease Control and Prev	Atlanta, GA, United States	Data collection	
Andrew	Moseray		MSc	Crown Agents	Makeni, Sierra Leone	Data collection	
Fatmata B	Tarawally		MSc	FOCUS 1000	Makeni, Sierra Leone	Data collection	
Martin	Seppeh		BSc	FOCUS 1000	Makeni, Sierra Leone	Data collection	
Ronald	Mash		DrPH	Ministry of Health and Sanitation, Fre	Freetown, Sierra Leone	Data collection	
Babatunde	Duduyemi		FMCPath	University of Sierra Leone Teaching H	Freetown, Sierra Leone	Data collection	
James	Bunn		MD	Human Development Team, British H	Freetown, Sierra Leone	Data collection	

Supplemental Online Content: Nonauthor Collaborators
*First name, last name, and suffix (if applicable) are required and will appear in PubMed.

*First Name and Middle Initial(s)	*Last Name	*Suffix (eg, Jr, III)	Academic Degrees	Institution	Location (city, state/province, country)	Role or Contribution, eg, chair, principal investigator	Group (if more than 1 Group listed in the byline) and/or Subgroup (eg, Steering Committee)
Alim	Swaray-Deen		FWACS - O	University of Sierra Leone Teaching H	Freetown, Sierra Leone	Data collection	
Joseph	Bangura		MPH	Ministry of Health and Sanitation, Fre	Freetown, Sierra Leone	Data collection	
Amara	Jambai		MSc	Ministry of Health and Sanitation, Fre	Freetown, Sierra Leone	Data collection	
Margaret	Mannah		MPH	Ministry of Health and Sanitation, Fre	Freetown, Sierra Leone	Data collection	
Okokon	Ita		FMCPath -	University of Calabar Teaching Hospit	Cross River, Nigeria	Data collection	
Cornell	Chukwuegbo		FMCPath -	Federal Medical Center/PathConsult	Cross River, Nigeria	Data collection	
Sulaiman	Sannoh		MD	St. Luke's University Health Network,	Easton, PA, United States o	Data collection	
Princewill	Nwajiobi		FMCPath –	National Hospital, Abuja, Nigeria	Abuja, Nigeria	Data collection	
Oluseyi	Balogun		MHM	Crown Agents	Makeni, Sierra Leone	Data collection	
Carrie J	Cain		RN	World Hope International, Makeni, Si	Makeni, Sierra Leone	Data collection	
Samuel	Pratt		MPH	FOCUS 1000	Makeni, Sierra Leone	Data collection	
Francis	Moses		Master of M	Ministry of Health and Sanitation, Fre	Freetown, Sierra Leone	Data collection	
Tom	Sesay			Ministry of Health and Sanitation, Fre	Freetown, Sierra Leone	Data collection	
James	Squire		MPhil Appl	Ministry of Health and Sanitation, Fre	Freetown, Sierra Leone	Data collection	
Joseph K	Sesay			Ministry of Health and Sanitation, Fre	Freetown, Sierra Leone	Data collection	
Osman	Kaykay		MMed in C	Ministry of Health and Sanitation, Fre	Freetown, Sierra Leone	Data collection	
Binyam	Halu		MPH	WHO	Freetown, Sierra Leone	Data collection	
Hailemariam	Legesse		Postgradua	UNICEF	Freetown, Sierra Leone	Data collection	
Francis	Smart			Ministry of Health and Sanitation, Fre	Freetown, Sierra Leone	Data collection	
Sartie	Kenneh			Ministry of Health and Sanitation, Fre	Freetown, Sierra Leone	Data collection	
Soter	Ameh		PhD	Crown Agents	Freetown, Sierra Leone	Data collection	
Fatima	Solomon		MD	South African Medical Research Council V	Johannesburg, South Africa	Data collection	
Gillian	Sorour		MD	Wits Health Consortium	Johannesburg, South Africa	Data collection	
Hennie	Lombaard		MD	University of Witwatersrand, Johannesbu	Johannesburg, South Africa	Data collection	
Jeannette	Wadula		MD	National Health Laboratory Service, Depa	Johannesburg, South Africa	Data collection	
Karen	Petersen		MD	Department of Paediatrics, Chris Hani Ba	Johannesburg, South Africa	Data collection	
Martin	Hale		MD	National Health Laboratory Service, Depa	Johannesburg, South Africa	Data collection	
Nelesh P	Govender		MD	National Institute for Communicable	Johannesburg, South Africa	Data collection	
Peter J	Swart		MD	National Health for Laboratory Service in	Johannesburg, South Africa	Data collection	
Sanjay G	Lala		MD	Department of Paediatrics and Perinatal	Johannesburg, South Africa	Data collection	
Richard	Chawana		PhD	South African Medical Research Council V	Johannesburg, South Africa	Data collection	

Supplemental Online Content: Nonauthor Collaborators
*First name, last name, and suffix (if applicable) are required and will appear in PubMed.

***First Name and Middle Initial(s)**	***Last Name**	***Suffix (eg, Jr, III)**	Academic Degrees	Institution	Location (city, state/province, country)	Role or Contribution, eg, chair, principal investigator	Group (if more than 1 Group listed in the byline) and/or Subgroup (eg, Steering Committee)
Amy	Wise		MSc	South African Medical Research Council Vaccines and Infectious Diseases Analytics Research Unit, University of the Witwatersrand, Johannesburg, South Africa	Johannesburg, South Africa	Data collection	
Nellie	Myburgh		PhD	South African Medical Research Council Vaccines and Infectious Diseases Analytics Research Unit, University of the Witwatersrand, Johannesburg, South Africa	Johannesburg, South Africa	Data collection	
