# Supplementary material for: Adherence to Perinatal Asphyxia or Sepsis Management Guidelines in Low- and Middle-Income Countries
Source: JAMA Netw Open. 2025 May 16;8(5):e2510790. doi: 10.1001/jamanetworkopen.2025.10790 (PMC12084843; doi:10.1001/jamanetworkopen.2025.10790)
Supplement: Supplement 3. — Data Sharing Statement [file jamanetwopen-e2510790-s003.pdf]

Data Sharing Statement

Rahman. Adherence to Perinatal Asphyxia or Sepsis Management Guidelines in Low- and Middle-Income Countries. *JAMA Netw Open*. Published May 16, 2025.
doi:10.1001/jamanetworkopen.2025.10790

Data
Data available: Yes
Data types: Deidentified participant data
How to access data: Access to the dataset can be made upon reasonable request to the corresponding author (chris.rees@emory.edu)
When available: With publication

Supporting Documents
Document types: Statistical/analytic code
How to access documents: Access to the code can be made upon reasonable request to the corresponding author (chris.rees@emory.edu)
When available: With publication

Additional Information
Who can access the data: Access to the dataset can be made upon reasonable request to the corresponding author (chris.rees@emory.edu)
Types of analyses: Secondary analyses, meta-analyses
Mechanisms of data availability: Access to the dataset can be made upon reasonable request to the corresponding author (chris.rees@emory.edu)
